# Supplementary material for: Comparative evaluation of EUCAST RAST and QuickMIC for rapid antimicrobial susceptibility testing of carbapenem-resistant organisms directly from positive blood cultures
Source: Microbiol Spectr. 2026 Jun 30;14(8):e03956-25. doi: 10.1128/spectrum.03956-25 (PMC13435937; doi:10.1128/spectrum.03956-25)
Supplement: Supplementary file 1 [file spectrum.03956-25-s0001.docx]

**Figure S1. Phylogenetic analysis of study isolates using core genome multi locus sequence typing (cgMLST).** (A) *E. coli*; (B) *K. pneumoniae*; (C*) P. aeruginosa*; (D) *A. baumannii*. Using WGS, data a circular phylogenetic tree was constructed based on the cgMLST results. Sequence types are colour coded, according to the legend. Additionally, colour codes are indicating the presence (+; green) of absence (-; grey) of relevant ARGs. Heatmap rings are arranged from innermost to outermost as follows: bla*CTX-M*, bla*IMP*, bla*KPC*, bla*NDM*-1, bla*NDM*-5, bla*OXA*-23-like, bla*OXA*-24-like, bla*OXA*-48-like, bla*OXA*-58-like, bla*PER*-1, bla*SHV*, bla*VEB*-1, *blaVIM*.

B

**Figure S2: Heat map of Boruta analysis for different antibiotics** Boruta importance analysis of discrepancies of each gene cluster with regard to categorical agreement for (A) QuickMIC and (B) EUCAST RAST (all time points combined).
