# Supplementary material for: Comparative evaluation of EUCAST RAST and QuickMIC for rapid antimicrobial susceptibility testing of carbapenem-resistant organisms directly from positive blood cultures
Source: Microbiol Spectr. 2026 Jun 30;14(8):e03956-25. doi: 10.1128/spectrum.03956-25 (PMC13435937; doi:10.1128/spectrum.03956-25)
Supplement: Supplementary file 2 [file spectrum.03956-25-s0002.docx]

**Table S1 Broth Microdilution plate measurement range and corresponding EUCAST breakpoints for Enterobacterales*, P. aeruginosa* and *A. baumanii***

| Antibiotic | | BMD measuring range (mg/L) | EUCAST breakpoints (v15)  (Enterobacterales, S/R) | EUCAST breakpoints (v15)  (*P. aeruginosa*, S/R) | EUCAST breakpoints (v15)  *Acinetobacter species*, S/R) |
| --- | --- | --- | --- | --- | --- |
| CTA | Cefotaxime | 0.125-8 | 1/2 | - | - |
| CTV | Ceftazidime/Avibactam | 0.25-16 | 8/8 | 8/8 | - |
| CTZ | Ceftazidime | 0.25-16 | 1/4 | 0.001/8 | - |
| MER | Meropenem | 0.25-16 | 2/8 | 2/8 | 2/8 |

**Table S2 – Overview of AST results for BMD (MIC and categorization), QuickMIC (MIC and categorization) all RAST timepoints (categorization) and time to result for QuickMIC for each strain and each antibiotic.**

|  | **BMD_mic** | **BMD_cat** | **QM_mic** | **QM_cat** | **RAST4h** | **RAST6h** | **RAST8h** | **RAST16-20h** | **time_QuickMIC** |
| --- | --- | --- | --- | --- | --- | --- | --- | --- | --- |
| CTA - *E. coli* | | | | | | | | | |
| QRC006 | >8 | R | ≥8.0 | R | R | R | R | R | 3h50min |
| QRC008 | >8 | R | ≥8.0 | R | R | R | R | R | 3h50min |
| QRC019 | >8 | R | ≥8.0 | R | R | R | R | R | 3h50min |
| QRC021 | >8 | R | ≥8.0 | R | R | R | R | R | 3h50min |
| QRC027 | >8 | R | ≥8.0 | R | R | R | R | R | 3h50min |
| QRC032 | >8 | R | ≥8.0 | R | R | R | R | R | 3h50min |
| QRC053 | >8 | R | ≥8.0 | R | R | R | R | R | 3h50min |
| QRC081 | >8 | R | ≥8.0 | R | R | R | R | R | 3h50min |
| QRC082 | >8 | R | ≥8.0 | R | R | R | R | R | 3h50min |
| QRC083 | >8 | R | ≥8.0 | R | R | R | R | R | 3h50min |
| QRC084 | >8 | R | 4.0 | R | R | R | R | R | 3h30min |
| QRC085 | >8 | R | ≥8.0 | R | R | R | R | R | 3h50min |
| QRC086 | >8 | R | ≥8.0 | R | R | R | R | R | 3h50min |
| QRC088 | >8 | R | ≥8.0 | R | R | R | R | R | 3h50min |
| QRC089 | >8 | R | ≥8.0 | R | R | R | R | R | 3h50min |
| QRC112 | >8 | R | ≥8.0 | R | R | R | R | R | 3h50min |
| QRC113 | >8 | R | ≥8.0 | R | R | R | R | R | 3h50min |
| QRC115 | >8 | R | 4.0 | R | R | R | R | R | 3h50min |
| QRC116 | >8 | R | ≥8.0 | R | R | R | R | R | 3h50min |
| QRC120 | >8 | R | ≥8.0 | R | R | R | R | R | 3h50min |
| QRC123 | >8 | R | 4.0 | R | ATU | R | R | R | 3h50min |
| QRC127 | >8 | R | ≥8.0 | R | R | R | R | R | 3h50min |
| QRC128 | >8 | R | ≥8.0 | R | R | R | R | R | 3h50min |
| QRC129 | >8 | R | ≥8.0 | R | R | R | R | R | 3h50min |
| CTA *- K. pneumoniae* | | | | | | | | | |
| QRC001 | >8 | R | ≥8.0 | R | R | R | R | R | 3h50min |
| QRC007 | >8 | R | ≥8.0 | R | R | R | R | R | 3h50min |
| QRC010 | >8 | R | ≥8.0 | R | R | R | R | R | 3h50min |
| QRC013 | >8 | R | - | - | R | R | R | R | - |
| QRC018 | >8 | R | ≥8.0 | R | R | R | R | R | 3h50min |
| QRC020 | >8 | R | ≥8.0 | R | R | R | R | R | 3h50min |
| QRC029 | >8 | R | ≥8.0 | R | R | R | R | R | 3h50min |
| QRC031 | >8 | R | ≥8.0 | R | R | R | R | R | 3h50min |
| QRC034 | >8 | R | ≥8.0 | R | R | R | R | R | 3h50min |
| QRC035 | >8 | R | ≥8.0 | R | R | R | R | R | 3h50min |
| QRC037 | >8 | R | ≥8.0 | R | R | R | R | R | 3h50min |
| QRC068 | >8 | R | ≥8.0 | R | R | R | R | R | 3h50min |
| QRC070 | >8 | R | ≥8.0 | R | R | R | R | R | 3h50min |
| QRC075 | >8 | R | ≥8.0 | R | R | R | R | R | 3h50min |
| QRC080 | >8 | R | ≥8.0 | R | R | R | R | R | 3h50min |
| QRC095 | >8 | R | ≥8.0 | R | R | R | R | R | 3h50min |
| QRC096 | >8 | R | ≥8.0 | R | R | R | R | R | 3h50min |
| QRC111 | >8 | R | ≥8.0 | R | R | R | R | R | 3h50min |
| QRC125 | >8 | R | ≥8.0 | R | R | R | R | R | 3h50min |
| QRC126 | >8 | R | - | - | R | R | R | R | - |
| QRC130 | >8 | R | 8.0 | R | R | R | R | R | 3h50min |
| QRC131 | >8 | R | ≥8.0 | R | R | R | R | R | 3h50min |
| CTZ - *E. coli* | | | | | | | | | |
| QRC006 | >16 | R | ≥16.0 | R | R | R | R | R | 3h50min |
| QRC008 | 2 | I | 8.0 | R | ATU | S | R | R | 3h20min |
| QRC019 | 8 | R | 8.0 | R | R | R | R | R | 3h50min |
| QRC021 | >16 | R | ≥16.0 | R | R | R | R | R | 3h50min |
| QRC027 | >16 | R | ≥16.0 | R | R | R | R | R | 3h50min |
| QRC032 | >16 | R | ≥16.0 | R | R | R | R | R | 3h50min |
| QRC053 | >16 | R | ≥16.0 | R | R | R | R | R | 3h50min |
| QRC081 | >16 | R | ≥16.0 | R | R | R | R | R | 3h50min |
| QRC082 | >16 | R | ≥16.0 | R | R | R | R | R | 3h50min |
| QRC083 | >16 | R | ≥16.0 | R | R | R | R | R | 3h50min |
| QRC084 | >16 | R | ≥16.0 | R | R | R | R | R | 3h50min |
| QRC085 | >16 | R | ≥16.0 | R | R | R | R | R | 3h50min |
| QRC086 | >16 | R | ≥16.0 | R | R | R | R | R | 3h50min |
| QRC088 | >16 | R | ≥16.0 | R | R | R | R | R | 3h50min |
| QRC089 | >16 | R | ≥16.0 | R | R | R | R | R | 3h50min |
| QRC112 | >16 | R | ≥16.0 | R | R | R | R | R | 3h50min |
| QRC113 | 8 | R | 8.0 | R | R | R | R | R | 3h20min |
| QRC115 | 2 | I | 0.5 | S | S | ATU | S | S | 3h20min |
| QRC116 | 8 | R | 8.0 | R | R | R | R | R | 3h10min |
| QRC120 | 4 | I | 8.0 | R | ATU | R | R | R | 3h0min |
| QRC123 | 16 | R | 8.0 | R | ATU | R | R | R | 3h50min |
| QRC127 | 8 | R | 8.0 | R | R | R | R | R | 3h50min |
| QRC128 | 16 | R | - | - | R | R | R | R | - |
| QRC129 | >16 | R | ≥16.0 | R | R | R | R | R | 3h50min |
| CTZ - *K. pneumoniae* | | | | | | | | | |
| QRC001 | >16 | R | ≥16.0 | R | R | R | R | R | 3h50min |
| QRC007 | 16 | R | ≥16.0 | R | R | R | R | R | 3h50min |
| QRC010 | >16 | R | ≥16.0 | R | R | R | R | R | 3h50min |
| QRC013 | >16 | R | ≥16.0 | R | R | R | R | R | 3h50min |
| QRC018 | 8 | R | 8.0 | R | R | R | R | R | 3h50min |
| QRC020 | 8 | R | 8.0 | R | R | R | R | R | 3h50min |
| QRC029 | >16 | R | ≥16.0 | R | R | R | R | R | 3h50min |
| QRC031 | >16 | R | ≥16.0 | R | R | R | R | R | 3h50min |
| QRC034 | >16 | R | ≥16.0 | R | R | R | R | R | 3h50min |
| QRC035 | >16 | R | ≥16.0 | R | R | R | R | R | 3h50min |
| QRC037 | >16 | R | ≥16.0 | R | R | R | R | R | 3h50min |
| QRC068 | >16 | R | ≥16.0 | R | R | R | R | R | 3h50min |
| QRC070 | >16 | R | ≥16.0 | R | R | R | R | R | 3h50min |
| QRC075 | >16 | R | ≥16.0 | R | R | R | R | R | 3h50min |
| QRC080 | >16 | R | ≥16.0 | R | R | R | R | R | 3h50min |
| QRC095 | >16 | R | ≥16.0 | R | R | R | R | R | 3h50min |
| QRC096 | >16 | R | ≥16.0 | R | R | R | R | R | 3h50min |
| QRC111 | >16 | R | ≥16.0 | R | R | R | R | R | 3h50min |
| QRC125 | >16 | R | ≥16.0 | R | R | R | R | R | 3h50min |
| QRC126 | >16 | R | ≥16.0 | R | R | R | R | R | 3h50min |
| QRC130 | >16 | R | ≥16.0 | R | R | R | R | R | 3h50min |
| QRC131 | >16 | R | ≥16.0 | R | R | R | R | R | 3h50min |
| CTZ *- P. aeruginosa* | | | | | | | | | |
| QRC011 | >16 | R | 16.0 | R | - | R | R | R | 3h50min |
| QRC015 | >16 | R | 16.0 | R | - | - | - | R | 3h50min |
| QRC042 | >16 | R | 16.0 | R | - | R | R | ATU | 3h50min |
| QRC044 | >16 | R | ≥16.0 | R | - | R | R | R | 3h50min |
| QRC045 | >16 | R | ≥16.0 | R | - | - | R | R | 3h50min |
| QRC046 | 16 | R | ≥16.0 | R | - | - | R | R | 3h50min |
| QRC047 | 16 | R | ≥16.0 | R | - | - | R | R | 3h50min |
| QRC048 | >16 | R | ≥16.0 | R | - | - | ATU | R | 3h50min |
| QRC049 | 8 | I | 16.0 | R | - | - | - | R | 3h50min |
| QRC050 | 8 | I | ≥16.0 | R | - | - | - | I | 3h50min |
| QRC051 | >16 | R | - | - | - | R | R | R | - |
| QRC054 | >16 | R | - | - | - | I | ATU | R | - |
| QRC055 | >16 | R | ≥16.0 | R | - | R | R | R | 3h50min |
| QRC056 | >16 | R | - | - | - | - | R | R | - |
| QRC065 | >16 | R | ≥16.0 | R | - | R | R | R | 3h50min |
| QRC067 | 8 | I | ≥16.0 | R | - | R | R | R | 3h50min |
| QRC072 | 16 | R | ≥16.0 | R | - | ATU | ATU | R | 3h50min |
| QRC074 | 8 | I | 8.0 | I | - | ATU | ATU | I | 3h50min |
| QRC077 | >16 | R | ≥16.0 | R | - | R | R | R | 3h50min |
| QRC079 | >16 | R | ≥16.0 | R | - | R | R | R | 3h50min |
| QRC092 | >16 | R | ≥16.0 | R | - | R | R | R | 3h50min |
| QRC094 | 8 | I | 16.0 | R | - | R | ATU | I | 3h50min |
| QRC100 | >16 | R | ≥16.0 | R | - | R | R | R | 3h50min |
| QRC118 | >16 | R | ≥16.0 | R | - | R | R | R | 3h50min |
| QRC124 | >16 | R | 16.0 | R | - | R | R | R | 3h50min |
| CTV - *E. coli* | | | | | | | | | |
| QRC006 | >16 | R | ≥16.0 | R | R | R | ATU | R | 3h50min |
| QRC008 | ≤0.25 | S | 0.5 | S | S | S | S | S | 3h10min |
| QRC019 | ≤0.25 | S | 0.5 | S | S | S | S | S | 3h0min |
| QRC021 | >16 | R | ≥16.0 | R | R | R | R | R | 3h50min |
| QRC027 | >16 | R | ≥16.0 | R | R | R | R | R | 3h50min |
| QRC032 | >16 | R | ≥16.0 | R | R | ATU | ATU | R | 3h50min |
| QRC053 | >16 | R | ≥16.0 | R | R | R | R | R | 3h50min |
| QRC081 | >16 | R | ≥16.0 | R | R | R | R | R | 3h50min |
| QRC082 | >16 | R | - | - | R | R | R | R | - |
| QRC083 | >16 | R | ≥16.0 | R | ATU | R | ATU | ATU | 3h50min |
| QRC084 | ≤0.25 | S | 0.5 | S | S | S | S | S | 3h20min |
| QRC085 | >16 | R | ≥16.0 | R | R | R | R | R | 3h50min |
| QRC086 | >16 | R | ≥16.0 | R | R | R | R | R | 3h50min |
| QRC088 | >16 | R | 16.0 | R | R | R | R | R | 3h50min |
| QRC089 | 2 | S | 2.0 | S | S | S | S | S | 3h40min |
| QRC112 | >16 | R | ≥16.0 | R | R | R | S | R | 3h50min |
| QRC113 | ≤0.25 | S | 0.5 | S | S | S | S | S | 2h50min |
| QRC115 | ≤0.25 | S | 0.5 | S | S | S | S | S | 3h10min |
| QRC116 | ≤0.25 | S | 0.5 | S | S | S | S | S | 2h50min |
| QRC120 | ≤0.25 | S | 0.5 | S | S | S | S | S | 3h10min |
| QRC123 | ≤0.25 | S | 0.5 | S | S | S | S | S | 3h30min |
| QRC127 | ≤0.25 | S | 0.5 | S | ATU | S | S | S | 3h10min |
| QRC128 | ≤0.25 | S | 1.0 | S | S | S | S | S | 2h30min |
| QRC129 | >16 | R | ≥16.0 | R | R | R | R | R | 3h50min |
| CTV - *K. pneumoniae* | | | | | | | | | |
| QRC001 | 1 | S | - | - | S | S | S | S | - |
| QRC007 | 0.5 | S | 1.0 | S | S | S | S | S | 3h0min |
| QRC010 | 0.5 | S | 1.0 | S | S | R | S | ATU | 3h30min |
| QRC013 | 0.5 | S | 1.0 | S | S | S | S | S | 3h30min |
| QRC018 | 0.5 | S | 1.0 | S | S | S | S | S | 3h10min |
| QRC020 | 0.5 | S | 2.0 | S | S | S | S | S | 3h0min |
| QRC029 | 0.5 | S | 1.0 | S | R | R | R | S | 3h20min |
| QRC031 | 0.5 | S | 1.0 | S | S | S | S | S | 3h20min |
| QRC034 | ≤0.25 | S | 0.5 | S | S | S | S | S | 3h0min |
| QRC035 | 1 | S | 1.0 | S | ATU | S | S | S | 3h10min |
| QRC037 | >16 | R | ≥16.0 | R | R | R | R | R | 3h50min |
| QRC068 | 0.5 | S | 1.0 | S | R | S | S | S | 3h50min |
| QRC070 | 1 | S | 0.5 | S | S | S | S | S | 3h10min |
| QRC075 | 0.5 | S | 1.0 | S | S | S | S | S | 3h10min |
| QRC080 | >16 | R | ≥16.0 | R | R | R | R | R | 3h50min |
| QRC095 | >16 | R | ≥16.0 | R | R | R | R | R | 3h50min |
| QRC096 | >16 | R | ≥16.0 | R | R | R | R | R | 3h50min |
| QRC111 | 1 | S | 2.0 | S | ATU | S | S | S | 3h20min |
| QRC125 | 1 | S | 1.0 | S | S | S | S | S | 3h30min |
| QRC126 | >16 | R | ≥16.0 | R | R | R | R | R | 3h50min |
| QRC130 | 1 | S | 2.0 | S | S | S | S | S | 3h20min |
| QRC131 | >16 | R | ≥16.0 | R | R | R | R | R | 3h50min |
| CTV - *P. aeruginosa* | | | | | | | | | |
| QRC011 | 8 | S | ≥16.0 | R | - | R | R | R | 3h50min |
| QRC015 | >16 | R | 16.0 | R | - | - | - | R | 3h50min |
| QRC042 | 8 | S | ≥16.0 | R | - | R | R | R | 3h50min |
| QRC044 | 16 | R | ≥16.0 | R | - | R | R | R | 3h50min |
| QRC045 | 8 | S | 16.0 | R | - | - | R | R | 3h50min |
| QRC046 | 8 | S | 8.0 | S | - | - | R | ATU | 3h50min |
| QRC047 | 8 | S | ≥16.0 | R | - | - | R | R | 3h50min |
| QRC048 | 16 | R | ≥16.0 | R | - | - | ATU | R | 3h50min |
| QRC049 | 8 | S | 4.0 | S | - | - | - | R | 3h50min |
| QRC050 | 8 | S | ≥16.0 | R | - | - | R | R | 3h50min |
| QRC051 | 8 | S | ≥16.0 | R | - | R | R | R | 3h50min |
| QRC054 | 16 | R | 0.5 | S | - | R | R | R | 3h50min |
| QRC055 | >16 | R | ≥16.0 | R | - | R | R | R | 3h50min |
| QRC056 | >16 | R | - | - | - | - | R | R | - |
| QRC065 | 16 | R | ≥16.0 | R | - | R | R | R | 3h50min |
| QRC067 | 8 | S | 16.0 | R | - | R | R | R | 3h50min |
| QRC072 | 8 | S | 16.0 | R | - | R | R | R | 3h50min |
| QRC074 | 8 | S | 8.0 | S | - | R | ATU | ATU | 3h50min |
| QRC077 | >16 | R | ≥16.0 | R | - | R | R | ATU | 3h50min |
| QRC079 | >16 | R | ≥16.0 | R | - | R | R | R | 3h50min |
| QRC092 | >16 | R | ≥16.0 | R | - | R | R | R | 3h50min |
| QRC094 | 8 | S | 16.0 | R | - | - | R | ATU | 3h50min |
| QRC100 | >16 | R | ≥16.0 | R | - | R | R | R | 3h50min |
| QRC118 | >16 | R | ≥16.0 | R | - | R | R | R | 3h50min |
| QRC124 | >16 | R | ≥16.0 | R | - | R | R | R | 3h50min |
| MER - *E. coli* | | | | | | | | | |
| QRC006 | >16 | R | 8.0 | I | R | R | R | R | 3h50min |
| QRC008 | 0.5 | S | 0.5 | S | S | S | S | S | 3h50min |
| QRC019 | 1 | S | 0.5 | S | ATU | S | S | R | 3h30min |
| QRC021 | 16 | R | 4.0 | I | R | R | R | R | 3h50min |
| QRC027 | 2 | S | 0.5 | S | R | R | R | R | 3h30min |
| QRC032 | 1 | S | 0.5 | S | R | R | ATU | S | 3h40min |
| QRC053 | >16 | R | 8.0 | I | R | R | R | R | 3h50min |
| QRC081 | >16 | R | 8.0 | I | R | R | R | R | 3h50min |
| QRC082 | >16 | R | - | - | R | R | R | R | - |
| QRC083 | >16 | R | 4.0 | I | R | R | R | R | 3h50min |
| QRC084 | 2 | S | 0.5 | S | R | R | R | R | 3h30min |
| QRC085 | 16 | R | 1.0 | S | R | R | R | R | 3h50min |
| QRC086 | >16 | R | 4.0 | I | R | R | R | R | 3h50min |
| QRC088 | >16 | R | 8.0 | I | R | R | R | R | 3h50min |
| QRC089 | 1 | S | 0.5 | S | R | ATU | ATU | S | 3h50min |
| QRC112 | 8 | I | 2.0 | S | R | R | R | R | 3h50min |
| QRC113 | 1 | S | 0.5 | S | ATU | ATU | ATU | ATU | 3h50min |
| QRC115 | 0.5 | S | 0.5 | S | ATU | S | S | S | 3h10min |
| QRC116 | 0.5 | S | 0.5 | S | S | S | S | S | 2h40min |
| QRC120 | 0.5 | S | 0.5 | S | ATU | S | S | S | 3h0min |
| QRC123 | 4 | I | 2.0 | S | R | R | R | R | 3h50min |
| QRC127 | 4 | I | 2.0 | S | R | R | R | R | 3h50min |
| QRC128 | 1 | S | 0.5 | S | S | S | S | S | 3h40min |
| QRC129 | >16 | R | 4.0 | I | R | R | R | R | 3h50min |
| MER - *K. pneumoniae* | | | | | | | | | |
| QRC001 | 8 | I | ≥16.0 | R | R | R | R | R | 3h50min |
| QRC007 | 16 | R | 4.0 | I | R | R | R | R | 3h50min |
| QRC010 | >16 | R | ≥16.0 | R | R | R | R | R | 3h50min |
| QRC013 | >16 | R | ≥16.0 | R | R | R | R | R | 3h50min |
| QRC018 | 16 | R | ≥16.0 | R | R | R | R | R | 3h50min |
| QRC020 | 16 | R | ≥16.0 | R | R | R | R | R | 3h50min |
| QRC029 | 16 | R | ≥16.0 | R | R | R | R | R | 3h50min |
| QRC031 | 16 | R | ≥16.0 | R | R | R | R | R | 3h50min |
| QRC034 | 1 | S | 0.5 | S | S | ATU | ATU | ATU | 3h50min |
| QRC035 | 16 | R | ≥16.0 | R | R | R | R | R | 3h50min |
| QRC037 | >16 | R | 8.0 | I | R | R | R | R | 3h50min |
| QRC068 | >16 | R | ≥16.0 | R | R | R | R | R | 3h50min |
| QRC070 | 16 | R | ≥16.0 | R | R | R | R | R | 3h50min |
| QRC075 | 8 | I | ≥16.0 | R | R | R | R | R | 3h50min |
| QRC080 | >16 | R | ≥16.0 | R | R | R | R | R | 3h50min |
| QRC095 | 16 | R | - | - | R | R | R | R | - |
| QRC096 | 16 | R | ≥16.0 | R | R | R | R | R | 3h50min |
| QRC111 | 16 | R | 8.0 | I | R | R | R | R | 3h50min |
| QRC125 | >16 | R | ≥16.0 | R | R | R | R | R | 3h50min |
| QRC126 | >16 | R | 8.0 | I | R | R | R | R | 3h50min |
| QRC130 | 8 | I | 4.0 | I | R | R | R | R | 3h50min |
| QRC131 | >16 | R | 4.0 | I | R | R | R | R | 3h50min |
| MER - *P. aeruginosa* | | | | | | | | | |
| QRC011 | 16 | R | ≥16.0 | R | - | R | R | R | 3h50min |
| QRC015 | >16 | R | ≥16.0 | R | - | - | R | R | 3h50min |
| QRC042 | >16 | R | ≥16.0 | R | - | R | R | R | 3h50min |
| QRC044 | >16 | R | ≥16.0 | R | - | R | R | R | 3h50min |
| QRC045 | >16 | R | ≥16.0 | R | - | - | R | R | 3h50min |
| QRC046 | 16 | R | ≥16.0 | R | - | - | R | R | 3h50min |
| QRC047 | >16 | R | ≥16.0 | R | - | - | R | R | 3h50min |
| QRC048 | >16 | R | ≥16.0 | R | - | - | R | R | 3h50min |
| QRC049 | >16 | R | ≥16.0 | R | - | - | - | R | 3h50min |
| QRC050 | >16 | R | ≥16.0 | R | - | - | R | R | 3h50min |
| QRC051 | >16 | R | ≥16.0 | R | - | R | R | R | 3h50min |
| QRC054 | >16 | R | 8.0 | I | - | R | R | R | 3h50min |
| QRC055 | >16 | R | ≥16.0 | R | - | R | R | R | 3h50min |
| QRC056 | >16 | R | - | - | - | - | R | R | - |
| QRC065 | >16 | R | ≥16.0 | R | - | - | - | R | 3h50min |
| QRC067 | >16 | R | ≥16.0 | R | - | R | R | R | 3h50min |
| QRC072 | 16 | R | ≥16.0 | R | - | R | R | R | 3h50min |
| QRC074 | 16 | R | ≥16.0 | R | - | R | R | R | 3h50min |
| QRC077 | >16 | R | - | - | - | R | R | R | - |
| QRC079 | >16 | R | ≥16.0 | R | - | R | R | R | 3h50min |
| QRC092 | >16 | R | ≥16.0 | R | - | R | R | R | 3h50min |
| QRC094 | 16 | R | ≥16.0 | R | - | R | R | R | 3h50min |
| QRC100 | >16 | R | ≥16.0 | R | - | R | R | R | 3h50min |
| QRC118 | >16 | R | ≥16.0 | R | - | R | R | R | 3h50min |
| QRC124 | >16 | R | ≥16.0 | R | - | R | R | R | 3h50min |
| MER - *A. baumannii* | | | | | | | | | |
| QRC002 | >16 | R | - | - | R | R | R | R | - |
| QRC005 | >16 | R | ≥16.0 | R | R | R | R | R | 3h50min |
| QRC009 | >16 | R | - | - | R | R | R | R | - |
| QRC012 | >16 | R | ≥16.0 | R | R | R | R | R | 3h50min |
| QRC023 | >16 | R | ≥16.0 | R | R | R | R | R | 3h50min |
| QRC024 | 16 | R | ≥16.0 | R | R | R | R | R | 3h50min |
| QRC025 | >16 | R | ≥16.0 | R | R | R | R | R | 3h50min |
| QRC026 | >16 | R | ≥16.0 | R | R | R | R | R | 3h50min |
| QRC059 | >16 | R | ≥16.0 | R | R | R | R | R | 3h50min |
| QRC060 | >16 | R | - | - | R | R | R | R | - |
| QRC063 | >16 | R | ≥16.0 | R | R | R | R | R | 3h50min |
| QRC064 | >16 | R | ≥16.0 | R | R | R | R | R | 3h50min |
| QRC073 | >16 | R | - | - | R | R | R | R | - |
| QRC078 | >16 | R | - | - | R | R | R | R | - |
| QRC090 | >16 | R | ≥16.0 | R | R | R | R | R | 3h50min |
| QRC091 | >16 | R | ≥16.0 | R | R | R | R | R | 3h50min |
| QRC097 | >16 | R | ≥16.0 | R | R | R | R | R | 3h50min |
| QRC098 | >16 | R | ≥16.0 | R | - | R | R | R | 3h50min |
| QRC099 | >16 | R | ≥16.0 | R | R | R | R | R | 3h50min |
| QRC101 | >16 | R | ≥16.0 | R | R | R | R | R | 3h50min |
| QRC102 | >16 | R | ≥16.0 | R | R | R | R | R | 3h50min |
| QRC103 | >16 | R | ≥16.0 | R | R | R | R | R | 3h50min |
| QRC104 | >16 | R | ≥16.0 | R | R | R | R | R | 3h50min |
| QRC105 | >16 | R | ≥16.0 | R | R | R | R | R | 3h50min |
| QRC106 | 16 | R | ≥16.0 | R | R | R | R | R | 3h50min |
| QRC107 | >16 | R | ≥16.0 | R | R | R | R | R | 3h50min |
| QRC108 | >16 | R | 4.0 | I | R | R | R | R | 3h50min |
| QRC109 | >16 | R | ≥16.0 | R | R | R | R | R | 3h50min |
| QRC110 | >16 | R | ≥16.0 | R | R | R | R | R | 3h50min |
| QRC114 | >16 | R | ≥16.0 | R | R | R | R | R | 3h50min |

**Supplementary Table S3:**

**Overview of overall results, results in ATU and non-readable results for all antibiotics and species measured by QuickMIC and results of RAST split up into the different reading time points. n.a. = not applicable, due to no interpretation criteria by EUCAST.**

|  |  | ***E. coli*** | | | | | ***K. pneumoniae*** | | | | | ***P. aeruginosa*** | | | | ***A. baumannii*** | | | | |
| --- | --- | --- | --- | --- | --- | --- | --- | --- | --- | --- | --- | --- | --- | --- | --- | --- | --- | --- | --- | --- |
|  |  | QuickMIC | RAST 4h | RAST 6h | RAST 8h | RAST 16-20h | QuickMIC | RAST 4h | RAST 6h | RAST 8h | RAST 16-20h | QuickMIC | RAST 6h | RAST 8h | RAST 16-20h | QuickMIC | RAST 4h | RAST 6h | RAST 8h | RAST 16-20h |
| CTA | Result | 24 (100%) | 23 (95.8%) | 24 (100%) | 24 (100%) | 24 (100%) | 20 (90.9%) | 22 (100%) | 22 (100%) | 22 (100%) | 22 (100%) | n.a. | n.a. | n.a. | n.a. | n.a. | n.a. | n.a. | n.a. | n.a. |
|  | ATU | - | 1 (4.2%) | 0 (0%) | 0 (0%) | 0 (0%) | 0 (0%) | 0 (0%) | 0 (0%) | 0 (0%) | 0 (0%) | n.a. | n.a. | n.a. | n.a. | n.a. | n.a. | n.a. | n.a. | n.a. |
|  | Not readable | 0 (0%) | 0 (0%) | 0 (0%) | 0 (0%) | 0 (0%) | 2 (9.1%) | 0 (0%) | 0 (0%) | 0 (0%) | 0 (0%) | n.a. | n.a. | n.a. | n.a. | n.a. | n.a. | n.a. | n.a. | n.a. |
| CTZ | Result | 23 (95.8%) | 21 (87.5%) | 23 (95.8%) | 24 (100%) | 24 (100%) | 22 (100%) | 22 (100%) | 22 (100%) | 22 (100%) | 22 (100%) | 22 (88%) | 15 (60%) | 17 (68%) | 24 (96%) | n.a. | n.a. | n.a. | n.a. | n.a. |
|  | ATU | - | 3 (12.5%) | 1 (4.2%) | 0 (0%) | 0 (0%) | 0 (0%) | 0 (0%) | 0 (0%) | 0 (0%) | 0 (0%) | 0 (0%) | 2 (8%) | 5 (20%) | 1 (4%) | n.a. | n.a. | n.a. | n.a. | n.a. |
|  | Not readable | 1 (4.2%) | 0 (0%) | 0 (0%) | 0 (0%) | 0 (0%) | 0 (0%) | 0 (0%) | 0 (0%) | 0 (0%) | 0 (0%) | 3 (12%) | 8 (32%) | 3 (12%) | 0 (0%) | n.a. | n.a. | n.a. | n.a. | n.a. |
| CTV | Result | 23 (95.8%) | 22 (91.7%) | 23 (95.8%) | 21 (87.5%) | 23 (95.8%) | 21 (95.5%) | 20 (90.9%) | 22 (100%) | 22 (100%) | 21 (95.5%) | 24 (96%) | 16 (64%) | 21 (84%) | 21 (84%) | n.a. | n.a. | n.a. | n.a. | n.a. |
|  | ATU | - | 2 (8.3%) | 1 (4.2%) | 3 (12.5%) | 1 (4.2%) | 0 (0%) | 2 (9.1%) | 0 (0%) | 0 (0%) | 1 (4.5%) | 0 (0%) | 0 (0%) | 2 (8%) | 4 (16%) | n.a. | n.a. | n.a. | n.a. | n.a. |
|  | Not readable | 1 (4.2%) | 0 (0%) | 0 (0%) | 0 (0%) | 0 (0%) | 1 (4.5%) | 0 (0%) | 0 (0%) | 0 (0%) | 0 (0%) | 1 (4%) | 9 (36%) | 2 (8%) | 0 (0%) | n.a. | n.a. | n.a. | n.a. | n.a. |
| MER | Result | 23 (95.8%) | 20 (83.3%) | 22 (91.7%) | 21 (87.5%) | 23 (95.8%) | 21 (95.5%) | 22 (100%) | 21 (95.5%) | 21 (95.5%) | 21 (95.5%) | 23 (92%) | 16 (64%) | 23 (92%) | 25 (100%) | 25 (83.3%) | 29 (96.7%) | 30 (100%) | 30 (100%) | 30 (100%) |
|  | ATU | - | 4 (16.7%) | 2 (8.3%) | 3 (12.5%) | 1 (4.2%) | 0 (0%) | 0 (0%) | 1 (4.5%) | 1 (4.5%) | 1 (4.5%) | 0 (0%) | 0 (0%) | 0 (0%) | 0 (0%) | 0 (0%) | 0 (0%) | 0 (0%) | 0 (0%) | 0 (0%) |
|  | Not readable | 1 (4.2%) | 0 (0%) | 0 (0%) | 0 (0%) | 0 (0%) | 1 (4.5%) | 0 (0%) | 0 (0%) | 0 (0%) | 0 (0%) | 2 (8%) | 9 (36%) | 2 (8%) | 0 (0%) | 5 (16.7%) | 1 (3.3%) | 0 (0%) | 0 (0%) | 0 (0%) |
| Overall | Result | 93 (96.9%) | 86 (89.6%) | 92 (95.8%) | 90 (93.8%) | 94 (97.9%) | 84 (95.5%) | 86 (97.7%) | 87 (98.9%) | 87 (98.9%) | 86 (97.7%) | 69 (92%) | 47 (62.7%) | 61 (81.3%) | 70 (93.3%) | 25 (83.3%) | 29 (96.7%) | 30 (100%) | 30 (100%) | 30 (100%) |
|  | ATU | - | 10 (10.4%) | 4 (4.2%) | 6 (6.2%) | 2 (2.1%) | 0 (0%) | 2 (2.3%) | 1 (1.1%) | 1 (1.1%) | 2 (2.3%) | 0 (0%) | 2 (2.7%) | 7 (9.3%) | 5 (6.7%) | 0 (0%) | 0 (0%) | 0 (0%) | 0 (0%) | 0 (0%) |
|  | Not readable | 3 (3.1%) | 0 (0%) | 0 (0%) | 0 (0%) | 0 (0%) | 4 (4.5%) | 0 (0%) | 0 (0%) | 0 (0%) | 0 (0%) | 6 (8%) | 26 (34.7%) | 7 (9.3%) | 0 (0%) | 5 (16.7%) | 1 (3.3%) | 0 (0%) | 0 (0%) | 0 (0%) |
